# The Self-Liking Brain: A VBM Study on the Structural Substrate of Self-Esteem

**DOI:** 10.1371/journal.pone.0086430

**Published:** 2014-01-29

**Authors:** Dmitrij Agroskin, Johannes Klackl, Eva Jonas

**Affiliations:** Department of Psychology, University of Salzburg, Salzburg, Austria; University of Medicine & Dentistry of NJ - New Jersey Medical School, United States of America

## Abstract

Abundant evidence suggests that self-esteem is an important personality resource for emotion regulation in response to stressful experiences. It was thus hypothesized that the relative grey matter volume of brain regions involved in responding to and coping with stress is related to individual differences in trait self-esteem. Using structural magnetic resonance imaging of 48 healthy adults in conjunction with voxel-based morphometry and diffeomorphic anatomical registration using exponentiated lie algebra (VBM-DARTEL), positive associations between self-esteem and regional grey matter volume were indeed found in the anterior cingulate cortex (ACC), right lateral prefrontal cortex (LPFC), right hippocampus, and left hypothalamus. In addition, self-esteem positively covaried with grey matter volume in the right temporo-parietal junction (TPJ), which has been implicated in pride and theory of mind. The results suggest that persons with low self-esteem have reduced grey matter volume in brain regions that contribute to emotion/stress regulation, pride, and theory of mind. The findings provide novel neuroanatomical evidence for the view that self-esteem constitutes a vital coping resource.

## Introduction

Although self-esteem ranks among the most extensively studied constructs in behavioral science [1], its neuroanatomical basis is poorly understood. To our knowledge, there is no volumetric whole-brain investigation of self-esteem's structural substrate. In the present investigation, we used structural magnetic resonance imaging (MRI) and voxel-based morphometry (VBM) [2] with Diffeomorphic Anatomical Registration using Exponentiated Lie algebra (DARTEL) [3] to examine regional grey matter volume differences underlying variability in trait self-esteem of 48 healthy adults.

Self-esteem constitutes an affectively laden self-evaluation [1]. Levels of trait self-esteem reflect how individuals generally or most typically feel about themselves. Personality and social psychological research has comprehensively demonstrated that individual differences in self-esteem are relevant to a variety of health-related phenomena. For example, low self-esteem has been linked to negative affect [4] and heightened vulnerability to psychosocial stressors, as well as diverse affective disorders, including major depression, posttraumatic stress disorder (PTSD), and anxiety disorders [5]–[8]. High self-esteem, conversely, has been found to predict higher psychological well-being and a lower reactivity to stressful events [9]–[12]. These benefits of self-esteem may be at least partly attributable to superior emotion regulation and stress coping skills of persons with high self-esteem [13]–[16]. Self-esteem is accordingly considered an important coping resource in the coping or threat management literature [17], [18]. If self-esteem's salutogenic effects can be traced to the workings of certain brain regions, these regions may be similar to those involved in coping with threatening, stressful experiences. To sum up, given the protective effects of self-esteem against affective, stress-related disorders, as well as its beneficial role in emotion regulation, we expected levels of self-esteem to covary with relative grey matter volume in regions that are relevant to emotion regulation in response to stressful events, and are dysfunctional or structurally abnormal in patients with affective disorders. In the following sections, we review evidence germane to this reasoning.

In agreement with this line of argument, the sole published volumetric analysis of self-esteem to our knowledge has revealed that persons with low self-esteem possess reduced hippocampal volume [19], [20]. This may contribute to their lowered resilience, since reduced hippocampal volume has been implicated in vulnerability to stress [21]. However, Pruessner et al. [19] only examined whether differences in self-esteem covaried with regional volume in the hippocampus and the amygdala (using manual segmentation instead of VBM-DARTEL), without finding a relationship in the latter region. Whether self-esteem is associated with volume in other brain structures involved in coping with stressful events is thus unknown to date. Beside the hippocampus, whose reduced volume constitutes a risk factor for the development of stress-related psychopathology (i.e., PTSD) [22], there are some further regions that also play a role in adaptive responses to stress and threat.

It has been hypothesized that the cognitive control of affect is strongly supported by frontolimbic circuitry, involving interactions between prefrontal and cingulate control systems [23], [24], see also [25]. These affect-regulatory cognitive control processes may modulate emotion-generative systems, such as the amygdala [24]. In support of this view, neuroimaging studies have revealed activation of anterior cingulate cortex (ACC) and right lateral prefrontal cortex (rLPFC), as well as accompanying deactivation of the amygdala during coping with negative stimuli [26]–[29]. Moreover, inverse correlations were reported between activations in the ACC, rLPFC and self-reported negative affect during threat regulation [30], [31]. Thus, there is ample fMRI evidence implicating the ACC and rLPFC in adaptive affect regulation [23], [24].

Of particular importance to our investigation, there is also neuroanatomical evidence suggesting that the ACC may play a major role in emotion regulation. Reduced grey matter volume in the ACC has been found to underlie difficulty in regulating negative emotion [32]. Furthermore, there is evidence of decreased grey matter volume in the ACC in persons with a strong inclination to ruminate [33], which is an important mediator of the relationship between low self-esteem and depression [34]. Finally, trait levels of cognitive reappraisal – an emotion regulation strategy that is positively related to self-esteem [16] – are positively linked to ACC volume [35]. As a side comment, it is worthy of note that research on emotion regulation has distinguished different strategies of regulating affect – cognitive reappraisal and expressive suppression [36]. Whereas reappraisal involves reinterpreting the meaning of an event, and is considered adaptive, suppression involves inhibiting emotion-expressive behavior, and is considered maladaptive [16]. Self-esteem is positively associated with reappraisal and negatively with suppression [16]. Summing up, there is also neuroanatomical evidence that is suggestive of a relationship between pronounced skills in emotion regulation, such as reappraisal, and ACC volume.

Importantly, there is also evidence that the cognitive control of affect is modulated by trait self-esteem. For instance, Taylor et al. [10] found stronger ACC activation in response to threatening than non-threatening stimuli. This effect was particularly strong in persons with high self-esteem, suggesting that high reactivity to threat may precede successful threat regulation. Accordingly, high self-esteem persons also displayed lower cortisol reactivity (i.e., stress response), as well as higher right lateral prefrontal cortex (rLPFC) and lower amygdala activity during a threat regulation task. Taylor et al.'s [10] findings may thus be interpreted as indicating superior stress regulation skills of persons with high self-esteem, as reflected in higher activity of affect-regulatory brain regions, such as the ACC and prefrontal cortex.

It is important to note, however, that the specific systems within the ACC and prefrontal cortex that are involved in the cognitive control of emotion may depend upon the specific type of emotion and the specific kind of regulatory mechanism utilized [24], [37]. This caveat is important given that influential functional differentiations of the ACC have been proposed, such that its dorsal-caudal part may be more involved in cognitive processes [38] or emotion appraisal [39], and the ventral-rostral part may be more relevant to emotional processes [38] or emotion regulation [39]. In view of this caveat and the quite heterogeneous findings with respect to the specific ACC regions involved in affect regulation (e.g., dorsal ACC in [30]; ventral ACC in [29]), we discuss the entire ACC as potentially underlying self-esteem's positive implications for emotion/stress regulation, instead of restricting our investigation to a certain part of it.

In light of Mak et al.'s [32] finding that reduced grey matter volume is associated with difficulty in emotion regulation, it is conceivable that low self-esteem persons' heightened vulnerability to stress is attributable to structural abnormalities in frontolimbic circuitry, including the ACC, prefrontal cortex, and hippocampus. Given that low self-esteem characterizes diverse affective disorders [6], [7], it may be informative to consider neuroanatomical studies involving participants who suffer from low self-esteem-related disorders. Accordingly, reduced regional volumes have been meta-analytically determined in persons suffering from major depressive disorder [40]. More precisely, differences between patients and healthy controls were revealed in the ACC, hippocampus, and prefrontal cortex, whereas no differences appeared in the amygdala, thalamus, and total brain. This suggests that depressed individuals exhibit volumetric abnormalities particularly in those brain areas that are involved in adaptive emotion processing and stress regulation. This morphometric pattern is consistent with the findings from Pruessner et al. [19], who demonstrated reduced hippocampal volume in persons with low self-esteem, without finding a correlation with amygdalar volume. Similar results have been reported regarding PTSD and panic disorder patients in terms of decreased volume in hippocampal, anterior cingulate, and prefrontal regions [41]–[43]. It is important to note that low self-esteem appears to play a role in all these disorders. Panic disorder has been linked to lowered self-esteem along with depression and PTSD [6], [44], as well as bipolar disorder [45], which is also accompanied by lowered volume in cingulate, hippocampal, prefrontal, and hypothalamic regions [46]–[49]. Thus, there is converging evidence that brain regions involved in affect regulation are volumetrically reduced in individuals who suffer from affective disorders related to low self-esteem.

The ACC, LPFC, hippocampus, and hypothalamus have also been implicated in adaptive responses to stress by neurobiological research on the stress response in the brain [50], [51]. The stress response is directly regulated by the paraventricular nucleus of the hypothalamus (PVN), which is part of the hypothalamo-pituitary-adrenocortical (HPA) axis, and plays a central role in the release of stress-related hormones, such as cortisol [52]. PVN neurons are excited by the amygdala and inhibited by the hippocampus, whereby hippocampal inhibition of the PVN is mediated by a neuronal relay, including GABAergic neurons in the dorsomedial hypothalamus and the lateral hypothalamic area [53], [54]. Prefrontal and anterior cingulate regions have also been ascribed a role in GABAergic stress inhibition through negative feedback regulation of the HPA axis [53]–[55]. This is consistent with a large body of clinical research on the important role of GABA in dysfunctional stress regulation, which is typical for affective disorders related to low self-esteem [56], [57]. Numerous studies have found various indicators of deficient GABA neurotransmission in the ACC, LPFC, and hippocampus in individuals suffering from mood disorders [58]–[61]. Moreover, there is genetic research that is consistent with an association between GABA and self-esteem. This research is focused on the GAD1 gene that contributes to the production of glutamic acid decarboxylase (GAD), which synthesizes GABA [62]. GAD1 has been linked to a latent phenotype based on common genetic factors underlying neuroticism, depression, and anxiety disorders [63]. Given the overlap between the genetic underpinnings of self-esteem, neuroticism, and depression [64], it is conceivable that GAD1 and GABA play an important role in self-esteem, too.

Summing up, several different lines of research have attributed a major role to the ACC, LPFC, hippocampus, and hypothalamus in emotional self-regulation in response to stressful events. We therefore expected low levels of self-esteem to be accompanied by reduced grey matter volume in these brain regions. From the opposite perspective, individuals with high self-esteem should exhibit heightened volume in these neural structures. This reasoning is consistent with considering self-esteem an important coping resource [18], [65], and a predictor of adaptive emotion regulation strategies [13], [14], [16]. This is the first structural whole-brain examination of trait self-esteem, to our knowledge. Given that prior research has demonstrated the usefulness of volumetric analyses to the study of personality traits [66], [67], we aimed at complementing the abundant behavioral and fMRI research on self-esteem with a neuroanatomical investigation.

## Materials and Methods

### Ethics statement

The study was approved by the ethics committee of the University of Salzburg (“Ethikkommission der Universität Salzburg”). All participants signed informed consent, and could withdraw participation at any point, although no participant made use of this option.

### Participants

The sample consisted of 48 students from different academic disciplines (31 women) from the University of Salzburg, after excluding two participants with missing data. Participants were recruited by attendees of psychology undergraduate courses, who approached potential participants on the campus or used university mailing lists. Participants' mean age was 23.0 years (*SD* = 3.25, *Md* = 23.0, *range*: 19–37). None reported any history of neurological disorders or prior head trauma. They received course credits and a digital copy of their structural whole-head scan for participation.

### Self-esteem

After undergoing the structural MRI scan, participants were given a packet of questionnaires, which included the Rosenberg Self-Esteem Scale (RSES) [68] (German version [69]). This scale consists of ten items and is broadly used as a measure for global trait self-esteem. Example items are “On the whole, I am satisfied with myself” and “I feel I do not have much to be proud of” (recoded). 18 participants used a 4-point scale (from 1 = totally disagree to 4 = totally agree; Sample A) and 30 participants completed this scale using a 5-point Likert scale (from 1 = totally disagree to 5 = totally agree; Sample B), because the whole sample consisted of two subsamples that were run on different occasions and pooled for the purpose of the present analysis (mean *N*-weighted *α* = .84 for the pooled sample). Thus, the *same items* were used with two *different Likert scales* (1–4 and 1–5). The subsamples had the following descriptive statistics. Sample A: *M* = 3.41, *Md* = 3.5, *range*: 2.40–4.0, *SE* (*M*) = 0.11, *SD* = 0.48, *v* = −0.70, *SE* (*v*) = 0.54, *α* = .88. Sample B: *M* = 4.02, *Md* = 4.0, *range*: 2.60–5.0, *SE* (*M*) = 0.11, *SD* = 0.58, *v* = −0.71, *SE* (*v*) = 0.43, *α* = .81. The slight negative skewness found in both subsamples is consistent with prior research with nonclinical samples [70]. Self-esteem was normally distributed in both subsamples, Sample A: *z* = 0.90, *p* = 0.39, Sample B: *z* = 0.85, *p* = 0.47. Note that Sample B has previously been used in another publication that investigated different hypotheses from the present study [71].

In order to rule out that self-esteem in our sample was unusually high/low, we compared self-esteem scores in Sample A with self-esteem scores from two nonclinical German-speaking samples where the same self-esteem measure has been used [72]. *M*s and *SD*s were very similar across all samples. Sample 1 (*N* = 161): *M* = 3.33, *SD* = 0.48. Sample 2 (*N* = 64): *M* = 3.42, *SD* = 0.39. One-Sample *t*-tests comparing mean self-esteem between Sample A and the two samples were not significant, *t*s(17)<1.07, *p*s>.30. Comparing Sample B to a nonclinical English-speaking sample yielded very similar results, Sample (*N* = 225): *M* = 3.92, *SD* = 0.64 [73], One-Sample *t*-test: *t*(29) = 0.97, *p* = .34. Thus, the self-esteem distributions in our subsamples were typical for nonclinical samples.

To further test the typicality of our sample, we also compared our participants (only Sample A) to depressed psychiatric inpatients from different samples, expecting our participants to have higher self-esteem. Indeed, individuals suffering from depression exhibited strongly reduced self-esteem levels compared to our participants. Sample 1 (*N* = 28): *M* = 1.99, *SD* = 0.54, *t*(17) = 12.53, *p*<.001 [74]. Sample 2: (*N* = 34): *M* = 2.61, *SD* = 0.88, *t*(17) = 7.06, *p*<.001 [75]. Hence, our participants may represent a typical nonclinical sample with respect to global trait self-esteem.

To render the scales comparable across the subsamples, we standardized all values, after computing the mean scores. Since all relevant parameters were equal across both subsamples (all participants were recruited at the same university in the same way, and scanned in the same MRI scanner), pooling both subsamples should be unproblematic. Yet, we treated the subsample variable as a covariate in all analyses to rule out possible confounds.

### Scanning protocol

Imaging was performed on a 3-T Siemens Tim Trio scanner equipped with a 32-channel head coil. A whole-head high-resolution structural scan of each participant was performed using a T1-weighted MPRAGE sequence (FoV: 256 mm, slice thickness 1.2 mm, TR = 2300 ms, Flip angle 9°), resolution: 1×1×1.2 mm.

### Data processing

Structural MRI data were processed using Statistical Parametric Mapping (SPM8, Wellcome Department of Cognitive Neurology) implemented in MATLAB R2008a (version 7.6.0, Mathworks, Sherborn, MA). To ameliorate the registration of the MRI images, the DARTEL toolbox for SPM8 was used. All processing steps were performed exactly as suggested by Ashburner [76]. In short, the anatomic images were first manually reoriented so that the mm coordinate of the anterior commissure matched the origin (0, 0, 0), and the orientation approximated Montreal Neurological Institute (MNI) space. Next, T1-weighted images were classified into grey matter, white matter, and cerebrospinal fluid (CSF) using the ‘new-segment’ routine implemented in SPM8, which provides both the native space versions and DARTEL imported versions of the tissues. The DARTEL imported versions of grey and white matter were used to generate the flow fields (that encode the shapes), and a series of template images by use of the ‘DARTEL (create templates)’ routine. Hereby, the accuracy of inter-subject alignment is augmented by modeling the shape of each brain using millions of parameters (three parameters per voxel). DARTEL aligns grey and white matter simultaneously among the images. This is achieved by generating more and more reliable average template data, to which the data are iteratively aligned. The flow fields and the final template image created in the previous step are then utilized to create smoothed (10 mm Gaussian FWHM), modulated, spatially normalized, and Jacobian scaled grey matter images resliced to 1.5×1.5×1.5 mm voxel size in MNI space.

### Statistical analysis

Variability in the regional volume of grey matter was analyzed using voxel-wise statistical parametric mapping. An absolute threshold for masking of 0.2 was used. Global normalization was performed via proportional scaling, which means that the preprocessed data were divided by the total intracranial volume. Total intracranial volume was obtained by summing up the overall volumes of grey matter, white matter, and CSF, which were calculated by means of the MATLAB script “get_totals” provided by Ridgway [77]. A multiple regression analysis was performed with self-esteem as the predictor and age, gender, and subsample as covariates; the intercept was modeled as well.

Since the extensive use of overly stringent corrections for multiple comparisons in neuroimaging research has been criticized as a potential cause of Type II errors (i.e., false negatives) [78], clusters of heightened/reduced regional volume were considered significant at a height threshold of *t* = 3.29, *p*<0.001 (uncorrected) in conjunction with an extent threshold of *k* = 5 in the whole-brain analysis for *a priori* specified regions known to be involved in stress and emotion regulation (ACC, rLPFC, hypothalamus, hippocampus). For additional statistical rigor, small volume corrections (FWE-corrected at cluster-level in SPM8, *p*<0.05; following initial thresholding at *p*<0.001, uncorrected) were conducted within the regions of interest that were defined by use of the Automated Anatomical Labeling (AAL) brain atlas [79], applied with the Wake Forest University Pickatlas toolbox [80]. For regions of interest that are not explicitly defined in the AAL brain atlas (hypothalamus, rLPFC) small volume corrections were performed using 9–18 mm radius spheres centered on coordinates taken from prior research (see also [81] for a similar analytical strategy using VBM).

Specifically, in line with Ochsner and Gross [23], [37], the entire ACC (dorsal and ventral) was specified in the AAL brain atlas (where these regions are called anterior cingulate and middle cingulate, respectively). Likewise, the bilateral hippocampus was defined using the AAL brain atlas.

In contrast, the hypothalamus was defined through specific coordinates from the tuberal region of the hypothalamus, as prior research on stress regulation has specifically implicated the dorsomedial hypothalamus and the lateral hypothalamic area in GABAergic inhibition of the HPA axis [53], [54]. The hypothalamic region of interest included a 9 mm radius sphere centered on the (bilateral) dorsomedial hypothalamus coordinates (x = 3.2, y = −3.0, z = −12.0), as well as a 9 mm radius sphere around the (bilateral) lateral hypothalamic area coordinates (x = 6.6, y = −4.5, z = −11.4), taken from Baroncini et al. [82].

With regard to the rLPFC, an 18 mm radius sphere was centered on the mean coordinates of two rLPFC coordinates that predicted a decrease of negative affect during emotion regulation by cognitive reappraisal (x = 45, y = 36, z = 25) [31]. Activity in this particular region of the rLPFC has previously been linked to the regulation of negative emotion, sadness, and pain [83]–[85]. A relatively large sphere (18 mm) was chosen because the variability of the rLPFC coordinates implicated in emotion regulation is quite large in the neuroimaging literature, including ventral and dorsal regions of the rLPFC (see, e.g., [31]).

All other brain regions not specified *a priori* were examined at a threshold corrected for multiple comparisons (FWE-corrected at cluster-level, *p*<0.05; following initial thresholding at *p*<.001, uncorrected; 5-voxel minimum cluster size) in an exploratory whole-brain analysis. All coordinates are reported in Montreal Neurological Institute (MNI) format.

## Results

**Figure 1:**
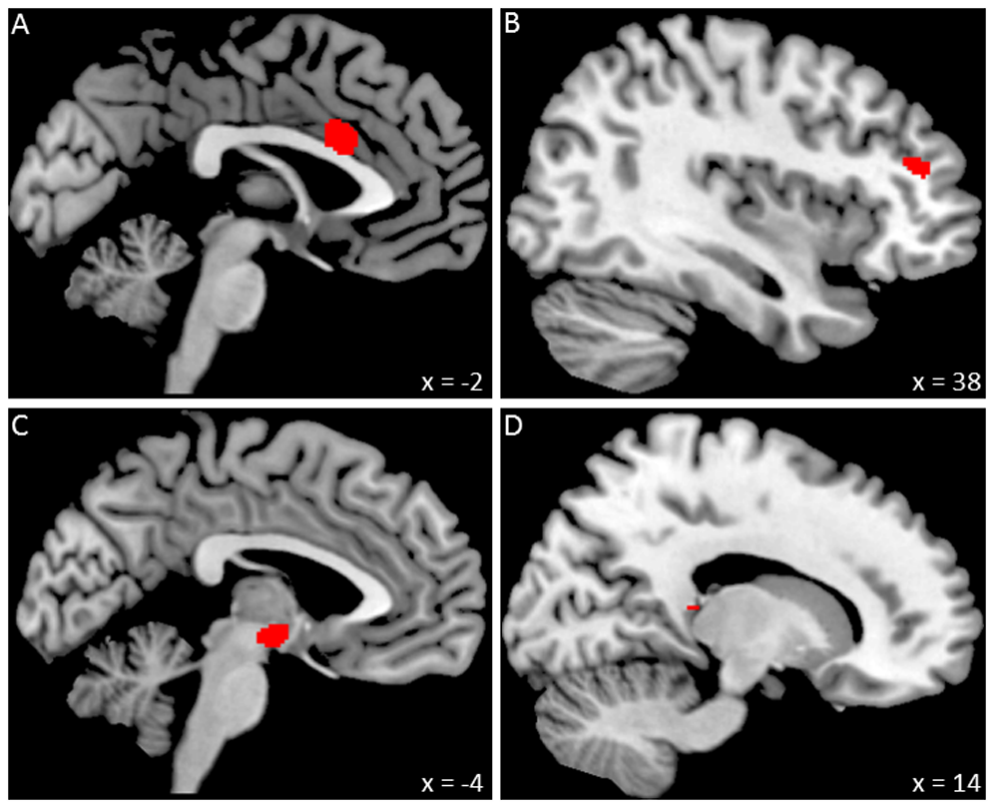
Positive associations between regional grey matter volume and individual differences in trait self-esteem. (A) anterior cingulate cortex, (B) lateral prefrontal cortex, (C) hypothalamus, (D) hippocampus (see also Table 1). Coordinates indicate the locations of the brain slices.

As hypothesized, trait self-esteem was positively related to relative grey matter volume in regions that have been associated with emotional self-regulation in response to stress, namely ACC, rLPFC, hypothalamus, and a small region in the right hippocampus (see Table 1 and Figure 1). All regions of interest survived small volume correction except the bilateral hippocampus (*p* = .079; one-tailed testing due to replication of [19]). Yet, defining only the right hippocampus as a region of interest in line with prior research on hippocampal volume and stress [86], resulted in a significant finding with regard to the hippocampus, too (*p* = .042; one-tailed). As indicated in Table 1, there were six voxels in the hippocampal cluster. According to the AAL brain atlas, two of the six voxels were outside the hippocampus. However, given that a similar location (x = 18, y = −32, z = 6) has been attributed to the hippocampus in prior stress research [87], we ascribe the entire cluster to the right hippocampus.

**Table 1:** Brain regions in which relative local grey matter volume was associated with trait self-esteem.

Anatomical region		*x*	*y*	*z*	*t*	*k*	*p*
TPJ	R	50	−58	24	4.49	647	<0.001
ACC*		0	17	24	4.30	352	<0.001
LPFC*	R	38	39	19	3.94	71	<0.001
Hypothalamus*	L	−2	−6	−8	3.92	254	<0.001
Hippocampus	R	14	−33	10	3.42	6	= 0.001

Regions of interest (ACC, LPFC, hypothalamus, hippocampus) are listed if they were significant at *p*<0.001 (uncorrected; 5-voxel minimum cluster size) in the whole-brain analysis. Regions of interest that survived small volume correction (FWE-corrected at cluster-level, *p*<.05; following initial thresholding at *p*<0.001, uncorrected) are indicated with an asterisk; the hippocampus marginally survived small volume correction (*p* = .079). The TPJ was not *a priori* hypothesized but was still significant after correction for multiple comparisons (FWE-corrected at cluster-level, *p*<0.05; following initial thresholding at *p*<0.001, uncorrected; 5-voxel minimum cluster size) in the whole-brain analysis. The individual *p*-values listed indicate the level of significance that each particular region met. R and L refer to right and left hemispheres; *x*, *y*, and *z* refer to MNI coordinates; *t* refers to the *t*-score at those coordinates (local maxima); *k* refers to the number of voxels in each significant cluster. The following abbreviations are used for the names of specific regions: temporoparietal junction (TPJ), anterior cingulate cortex (ACC), lateral prefrontal cortex (LPFC).

**Figure 2:**
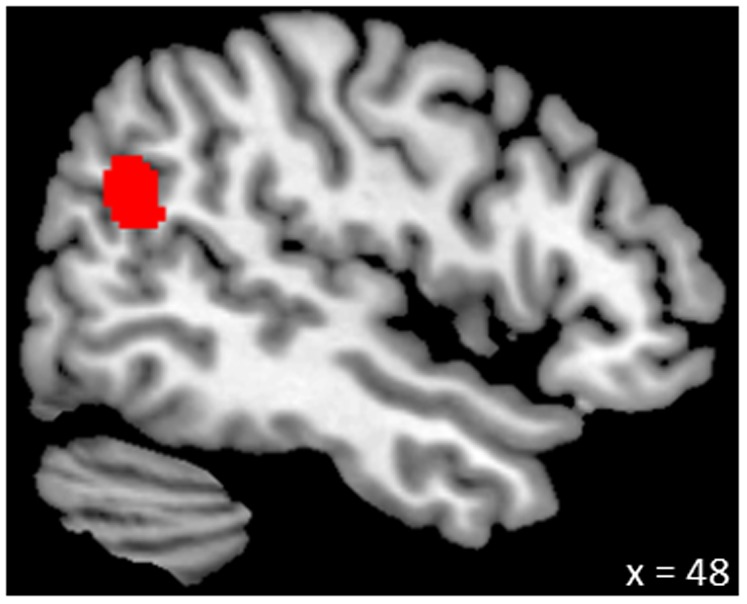
Positive association between grey matter volume in the right temporo-parietal junction and self-esteem. The coordinate indicates the location of the brain slice (see also Table 1).

Moreover, the exploratory whole-brain analysis revealed self-esteem to be positively linked to grey matter volume in the right temporo-parietal junction (rTPJ; Fig. 2). There were no regions in which self-esteem displayed a negative relationship with regional grey matter volume (after correcting for multiple comparisons in not *a priori* specified regions).

## Discussion

As predicted, the volumetric variability in certain brain structures was associated with individual differences in trait self-esteem. Persons with low self-esteem had reduced levels of regional grey matter volume in structures known to play a role in emotional self-regulation in response to threatening and stressful experiences. Our hypotheses – derived from functional and structural MRI research on emotion and stress regulation – were thus corroborated.

The results are consistent with abundant neurostructural research demonstrating neural atrophy in affective disorders (e.g., depression) [40], which are associated with low self-esteem [6] and emotion dysregulation [88]–[90]. Moreover, ample functional MRI research has implicated the very same brain structures in emotion/stress regulation [18], [23], which is something that low self-esteem persons are quite bad at doing [13], [14], [16]. In particular, our finding of heightened grey matter volume in a dorsal part of the ACC (x = 0, y = 17, z = 24) as a function of self-esteem approximates a region of the dorsal ACC (x = 6, y = 14, z = 32) that has been revealed to predict a drop in negative affect due to reappraisal in response to negative stimuli [30]. This result is also in line with neuroanatomical evidence relating the use of cognitive reappraisal – an especially adaptive emotion regulation strategy that is related to self-esteem [16] – to grey matter volume in the dorsal ACC [35]. In summary, the results are supportive of the view that self-esteem goes along with heightened grey matter volume in regions that are involved in adaptive responses to stressful events. This buttresses the widely accepted hypothesis that self-esteem is an important personality resource for stress and threat management [15], [17], [18].

Thus, the findings are consistent with the notion that the beneficial health effects of self-esteem may be linked to high self-esteem persons' pronounced skills in affect regulation [16]. Given that regional grey matter volume can increase as a function of the amount of training on a certain skill [91], high self-esteem persons' heightened regional volumes may reflect increased habitual use of emotion regulation strategies, such as stressor reappraisal [16]. Yet, this interpretation is fairly speculative and longitudinal research should shed more light on the question of causality, especially given that the regions found are not solely associated with emotion/stress regulation in terms of psychological functioning. It is important to note therefore that our cross-sectional neurostructural findings do not pertain to causal or functional relationships between self-esteem and psychological processes, such as emotion regulation [92].

It is furthermore noteworthy that the positive relationship of self-esteem and hippocampal volume replicates a prior finding of Pruessner et al. [19]. Yet, this link was rather small and only present in the right hippocampus in our sample. It has to be noted, though, that Pruessner et al. [19] utilized manual segmentation instead of VBM-DARTEL. The effect sizes are therefore difficult to compare. In addition, prior research on the association between chronic life stress and hippocampal volume in healthy persons solely found a negative relationship in the right hippocampus [86]. The overall similar results of the present work and Pruessner et al. [19] may speak to the reliability of the self-esteem-hippocampus relationship, though, especially given that they were obtained via different segmentation techniques.

Of particular interest may also be the unpredicted finding of strongly increased grey matter volume in the right TPJ (x = 50, y = −58, z = 24) among persons with high self-esteem. Notably, an fMRI study revealed positive correlations between self-ratings of pride and the degree of activation in this region (x = 44, y = −66, z = 20) [93]. Given that the inclination to experience pride is explicitly addressed by one self-esteem item (“I feel I do not have much to be proud of”; recoded), this finding may indicate that the rTPJ is involved in processes that give rise to positive self-evaluations.

According to social psychological theorizing, pride reflects self-esteem, joy, or pleasure derived from achievements, and arises from persons' positive inferences about others' appraisals of them, such as with positive feedback after performance tests [94]. This suggests that pride depends on mentalizing (i.e., thinking about other people's thoughts) in order to understand how one is evaluated by others. The rTPJ has been accordingly linked to theory of mind processes, specifically to thinking about others' thoughts [95], [96]. In support of a role of mentalizing in self-esteem, children's theory of mind skills have been revealed to be positively associated with their self-esteem [97]. It has also been proposed that mentalizing contributes to cognitive control of affect, since mental state attribution may be critical for comprehension and regulation of emotion [98]. It may not come as a surprise therefore that activation in the rTPJ (x = 54, y = −48, z = 34) has been found to predict a drop in negative affect due to reappraisal along with the dorsal ACC [30]. To sum up, deficient theory of mind skills may be linked to low self-esteem [97], and reduced grey matter volume in the rTPJ may be the neural substrate of this deficiency.

It may also be noteworthy that the rTPJ-related ability to reason about the contents of mental states seems to be uniquely human [99], contrary to more basic forms of social cognition, such as social interest and joint attention, which may be subserved by the ACC in humans [100], [101], as well as macaques [102], [103] and rats [104]. In evolutionary terms, self-esteem may thus depend upon both old (ACC) and new, that is, uniquely human (rTPJ) social cognitive mechanisms.

### Limitations and future research

Two limitations are important to mention. First, while our sample size should have been acceptable for our *a priori* tests involving small volume corrections (see, e.g., [81] for a VBM study involving a similar sample size and analytical strategy), it might have been rather low for the exploratory whole-brain examination, which only yielded the TPJ finding. Although sample sizes of around *N* = 50 are considered appropriate for whole-brain analyses [105], type II errors (false negatives) cannot be ruled out, implying that there may be further regions that are volumetrically related to self-esteem, but were rejected due to insufficient power. Future research might address this possibility.

Second, we did not control for personality traits that are related to self-esteem to obtain evidence for the specificity of the relationship between self-esteem and regional grey matter volumes. Trait reappraisal, for instance, has also been found to be positively related to dorsal ACC volume [35], suggesting that the associations found may be confounded by self-esteem's association with reappraisal. However, the specific combination of increased grey matter volumes in the ACC, right LPFC, left hypothalalmus, right hippocampus, and also right TPJ has not been linked to any other personality trait yet, to our knowledge. It may thus be rather unlikely that all of these relationships can be explained by another underlying factor, such as trait reappraisal. Even if self-esteem's relationship with dorsal ACC volume may be reducible to reappraisal, it may be difficult to explain why the same should be true for the relationship with, for example, rTPJ volume, especially given that this region appears to be specifically related to pride/self-esteem (controlling for joy) [93]. Another personality construct potentially confounding self-esteem's relationship with regional grey matter volume may be antisocial behavior, which is negatively related to self-esteem [106]. There is VBM evidence for reduced grey matter volume in the insula, orbitofrontal, and superior temporal cortex in individuals inclined to antisocial behaviors [107]. Interestingly, the superior temporal cortex partly overlaps with the TPJ, which is volumetrically reduced in persons with low self-esteem. This might suggest partly similar neuroanatomical underpinnings – possibly related to deficient theory of mind skills – for antisocial behavior and reduced self-esteem. However, the absence of volumetric associations between self-esteem and the insula and orbitofrontal cortex could be taken as evidence that a correlation between a given personality construct and self-esteem does not necessarily involve the same neural underpinnings. This is consistent with our reasoning that the findings may not be confounded by unmeasured personality correlates of self-esteem. However, future research may explicitly address this issue by including personality covariates in neurostructural investigations of self-esteem.

To conclude, our results are consistent with the hypothesis that persons with low self-esteem have reduced grey matter volume in brain regions known to support emotional self-regulation in response to stress. Moreover, the strong association with right TPJ volume may reflect high self-esteem individuals' inclination to experience feelings of pride [68], [108], as well as their superior theory of mind skills [97]. Hence, the findings provide novel neuroanatomical evidence for the view that dispositional self-esteem constitutes an important coping resource [18]. Given that regional grey matter volume appears to be malleable through training [91], psychological interventions designed to enhance the self may hold promise for promoting resilience to stressful events [15]. Based on our findings, interventions focused on self-esteem might be evaluated with MRI using the criterion of whether they increase grey matter volume in regions found to correlate with self-esteem in longitudinal designs.
